# In Vitro Photoinactivation of *Fusarium oxysporum* Conidia with Light-Activated Ammonium Phthalocyanines

**DOI:** 10.3390/ijms24043922

**Published:** 2023-02-15

**Authors:** Sara R. D. Gamelas, Isabel N. Sierra-Garcia, Augusto C. Tomé, Ângela Cunha, Leandro M. O. Lourenço

**Affiliations:** 1 LAQV-REQUIMTE, Department of Chemistry, University of Aveiro, 3810-193 Aveiro, Portugal; 2 CESAM, Department of Biology, University of Aveiro, 3810-193 Aveiro, Portugal

**Keywords:** antimicrobial photodynamic therapy (aPDT), *Fusarium oxysporum* conidia, cationic phthalocyanines, reactive oxygen species (ROS), singlet oxygen

## Abstract

Antimicrobial photodynamic therapy (aPDT) has been explored as an innovative therapeutic approach because it can be used to inactivate a variety of microbial forms (vegetative forms and spores) without causing significant damage to host tissues, and without the development of resistance to the photosensitization process. This study assesses the photodynamic antifungal/sporicidal activity of tetra- and octasubstituted phthalocyanine (Pc) dyes with ammonium groups. Tetra- and octasubstituted zinc(II) phthalocyanines (**1** and **2**) were prepared and tested as photosensitizers (PSs) on *Fusarium oxysporum* conidia. Photoinactivation (PDI) tests were conducted with photosensitizer (PS) concentrations of 20, 40, and 60 µM under white-light exposure at an irradiance of 135 mW·cm^–2^, applied during 30 and 60 min (light doses of 243 and 486 J·cm^−2^). High PDI efficiency corresponding to the inactivation process until the detection limit was observed for both PSs. The tetrasubstituted PS was the most effective, requiring the lowest concentration and the shortest irradiation time for the complete inactivation of conidia (40 µM, 30 min, 243 J·cm^−2^). Complete inactivation was also achieved with PS **2**, but a longer irradiation time and a higher concentration (60 µM, 60 min, 486 J·cm^−2^) were necessary. Because of the low concentrations and moderate energy doses required to inactivate resistant biological forms such as fungal conidia, these phthalocyanines can be considered potent antifungal photodynamic drugs.

## 1. Introduction

The *Fusarium* genus corresponds to a filamentous fungus commonly found in soil and plants, and it includes pathogen species to plants, animals, and humans [1]. *Fusarium* diseases affect many crop plants, imposing significant economic losses on fruit, vegetable, cereal, and cellulose production [2,3]. In humans, *Fusarium oxysporum* is an opportunistic pathogen that causes keratitis, onychomycosis, and invasive infection in immunocompromised and immunocompetent patients [4,5].

The asexual life cycle of *F. oxysporum* involves the production of chlamydospores, macroconidia, and microconidia, which ensures highly efficient dissemination in the environment [6]. Plant infection occurs through the roots. After the dispersal of conidia by wind or rain, germination begins in rhizosphere soil and growing hyphae penetrate root tissues, initiating infection [6,7]. Conventional fungicides usually target the conidial germination and early development stages [8].

In order to control infection by *Fusarium* species, the most effective choice is the use of resistant plant varieties. However, the tolerance level depends on the conditions in which they grow. In some regions, even with the use of resistant plant varieties, if the temperatures are elevated, the colonization by *Fusarium* spp. can be severe [9]. Besides the use of resistant plant varieties, some of the applied strategies are the use of seeds and seedlings already treated with fungicides before planting, and the use of fungicides through crop development [9,10]. However, the rise of tolerant fungal strains renders these strategies unreliable. The intensive use of the current fungicides is also considered a potential risk to humans and the environment [11,12]. To control the possibility of fungal inoculum in postharvest crops, it is common to treat them with chlorine or organic acids, even though they can be toxic to the environment [13].

The increased development of resistance to currently used fungicides and the progressive ban of the most popular pesticides in the EU imposes a drastic limitation on the chemical options to control fungal diseases in crops [14], so developing more effective technologies to control pathogenic fungi has gained interest. Interest in antimicrobial photodynamic therapy (aPDT) as an alternative for the inactivation of microorganisms in environmental matrices has been growing [15,16]. In fact, it was successfully used to inactivate or kill bacteria or fungi in animal hosts and environments [17,18,19,20,21]. aPDT is based on three nontoxic elements: a photosensitizer (PS), visible light, and oxygen (^3^O_2_). The combination of these three elements generates reactive oxygen species (ROS, e.g., singlet oxygen (^1^O_2_) and free radicals) that are responsible for the lethal oxidative damage of microbial targets (lipids, proteins, and nucleic acids), leading to the death of the target cells without causing significant damage to the host cells [22,23].

The application of aPDT for plant pathogens represents one of the latest developments for this technique aiming at alternatives to toxic agrochemicals. It was efficient in controlling bacterial diseases such as kiwifruit cancer [24] and citrus cancer [25]. This therapy was also used successfully against phytopathogenic fungi such as *Lasiodiplodia theobromae* (causes vine trunk disease), *Botrytis cinerea* (causes plant necrosis), and *Colletotrichum* sp. (causes anthracnose in various fruit trees) [26,27,28]. As already mentioned, spores are crucial to spreading fungal diseases, and fungal conidia are relevant targets for photosensitization [29].

Studying the structure–activity relationship is crucial in designing powerful PSs capable of inducing lethal damage to plant pathogens within short irradiation periods while preserving the integrity of host plant tissues. Porphyrin (Por) [21,30,31,32,33,34], chlorin (Chl) [16,35,36], and phthalocyanine (Pc) [37,38,39] dyes have been extensively used for the aPDT approach. Pcs display unique UV-vis spectra, typically with a Soret band at a wavelength maximum of 350 nm and intense Q bands in the red/near-IR region (600–800 nm) [37,39,40]. Structural features and particular functionalities greatly determine the physicochemical properties and biological activities of Pcs. The modification of the Pc macrocycle with ammonium peripheral moieties or the introduction of metallic ions (e.g., Zn(II)), which may improve the triplet excited state features and the ^1^O_2_ quantum yield, is considered a reliable strategy to finetune the physicochemical properties of Pcs in relation to the intended microbial targets [37,39].

Regarding fungal spores, the affinity of a PS is affected by the overall hydrophobicity of the spore coating and by the charged units in the PS structure [41]. So, a PS might continue to be adsorbed to the outer layers of spores (or vegetative hyphae) or reach the intercellular compartment. In this case, there is an increase in the spectra of both physiological and biochemical targets [26,42]. Fungal conidia have eukaryotic genomes and are less susceptible to oxidative stress than prokaryote cells are, but they have a more comprehensive array of subcellular targets. Therefore, if there is a multitarget photosensitization capacity of a PS, there is a reduced ability of these spores to develop resistance [41].

This work assesses the antifungal photodynamic activity of two quaternized zinc(II) Pc derivatives, and determines the relations between the number of cationic peripheral substituents using (**1**) tetra- and (**2**) octasubstituted ammonium phthalocyanines [39].

## 2. Results

### 2.1. Synthesis and Photophysical Characterization of Phthalocyanine Derivatives

**Figure 1:**
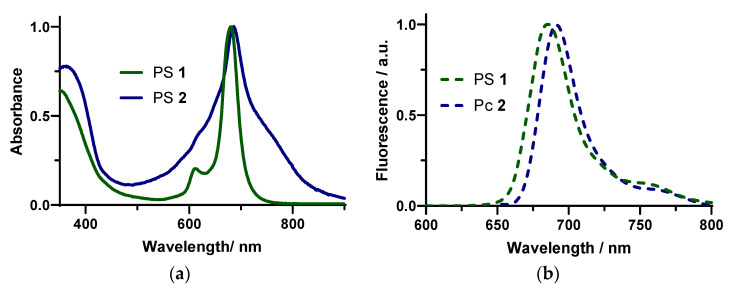
Normalized (**a**) absorption and (**b**) emission spectra of compounds **1** and **2** in DMF at 298 K (λ_exc_ PS **1** = 680 nm and λ_exc_ PS **2** = 643 nm).

PS **1** and **2** were synthesized and characterized by NMR (Figure S1–S3) according to the literature [39,43]. The absorption and emission spectra of **1** and **2** were determined in *N*,*N*-dimethylformamide (DMF) at low concentrations (10^−6^ M) [39]. The absorption spectra (Figure 1a) show the characteristic absorption features of zinc (II) Pcs with a high absorption band in the range of 300–450 nm (Soret band), and strong Q bands between 630 and 750 nm. Considering the excited state, after excitation at different wavelengths, both ZnPcs showed an emission band with the maximum between 670–691 nm (Figure 1b). The fluorescence quantum yields (Φ_F_) in DMF were less than 0.01 [39].

Given the potential use of PSs **1** and **2** as agents against *Fusarium oxysporum* conidia, it was essential to assess their ability to generate singlet oxygen. A previous assessment of the ^1^O_2_ generation capacity of these Pc derivatives, as determined with the indirect method of the 9,10-dimethylanthracene (9,10-DMA) absorption decay, showed that the tetrasubstituted PS **1** (Φ_Δ_ = 0.14) produced more ^1^O_2_ than the fluorinated octasubstituted PS **2** did (Φ_Δ_ = 0.03) [39].

### 2.2. Photodynamic Inactivation of Fusarium oxysporum Conidia

**Figure 2:**
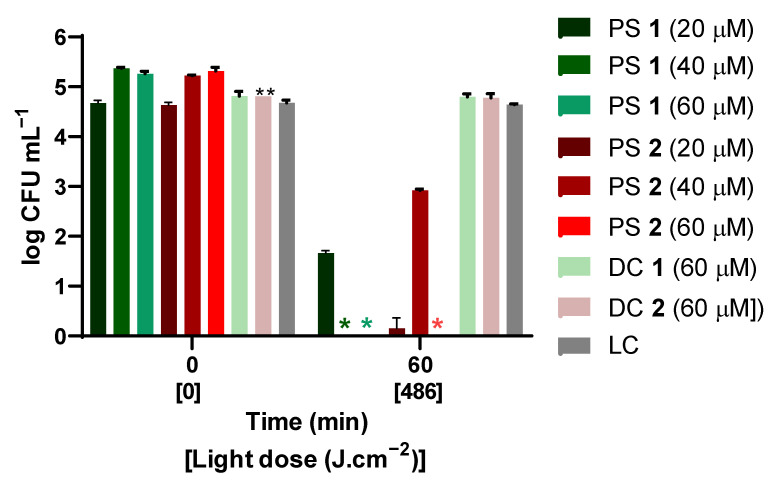
Variation in the concentration of viable conidia of *F. oxysporum* after 60 min irradiation with artificial white light at a fluence rate of 135 mW·cm^−2^ (486 J·cm^−2^) in the presence of 20, 40, and 60 μM of PS **1** or PS **2**. LC, light control; DC **1**, dark control with PS **1** at 60 μM; DC **2**, dark control with PS **2** at 60 μM. Values correspond to the average of three independent experiments with replicates. Error bars represent the standard deviation. *, no colonies observed; **, only one assay with three analytic replicates in which the number of colonies was exactly the same, producing a STD value of 0.

Figure 2 shows the logarithmic reduction in the concentration of viable *F. oxysporum* conidia after 60 min of irradiation with artificial white light at a fluence rate of 135 mW·cm^−2^ (light dose of 486 J·cm^−2^) in the presence of PS **1** and PS **2** at 20, 40, and 60 μM. After 60 min of irradiation, the highest concentration (60 μM) of either PS **1** or PS **2** caused a significant reduction in the concentration of viable *F. oxysporum* conidia. At the highest tested concentration (60 µM), none of the PSs caused lethal damage in the absence of light (dark controls: DC PS **1** and DC PS **2**, Figure 2). Moreover, PS **1** caused the complete inactivation of conidia (>5 log_10_ reduction) upon 60 min of irradiation at both the tested concentrations (40 and 60 µM). Within the light dose, a lower concentration of 20 μM of PS **1** caused only a 2 log_10_ reduction in conidia viability. PS **2** caused a steady decrease in the viability of conidia with increasing concentrations: reduction of 2, 3, and 5 log_10_ with 20, 40, or 60 µM, respectively, at 60 min of light irradiation.

**Figure 3:**
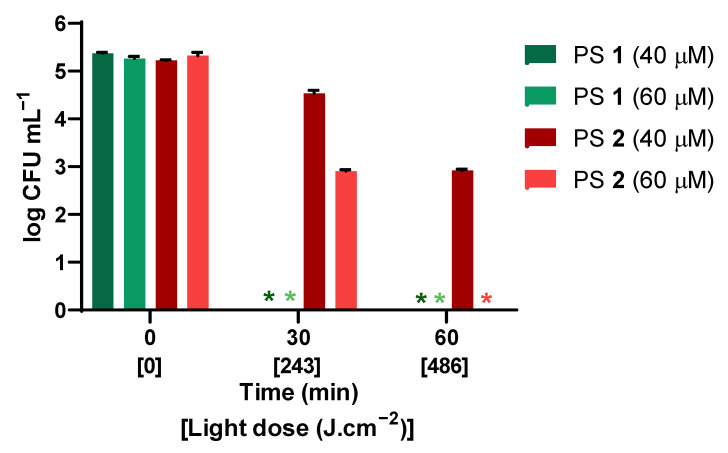
Variation in the concentration of viable conidia of *F. oxysporum* in the presence of 40 and 60 μM of PS **1** or PS **2** exposed to white light for 0, 30, and 60 min (135 mW·cm^−2^). Values are the mean of three replicate counts. Error bars represent the standard deviation. *, there was no colony present.

To understand the inactivation kinetics with PS **1** or **2**, the viability of conidia after 30 and 60 min of irradiation with white light was determined as represented in Figure 3. PS **1** was more effective than PS **2**, as either 40 or 60 μM caused the complete inactivation of *F. oxysporum* conidia within 30 min of irradiation. On the other hand, PS **2**, at the highest tested concentration (60 μM), showed a reduction of approximately 2 logs in the concentration of viable conidia after 30 min of irradiation, and complete inactivation after 60 min. No significant inactivation was evident with 40 μM of PS **2** after 30 min of irradiation, but after 60 min of irradiation, a reduction of 2 logs was observed.

## 3. Discussion

**Figure 4:**
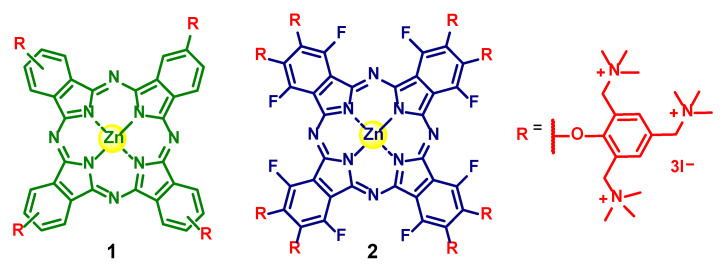
Structures of tetra- and octasubstituted zinc(II) phthalocyanines **1** and **2**.

The development of the resistance of conidia to chemical and physical fungal agents motivated research for new effective, sustainable, and environmentally friendly methods for their control, such as the aPDT approach. The structure of PS molecules is one of the major determinants of the efficiency of photosensitization [37,44,45,46]. In particular, positive charges are essential to make the PSs more water-soluble and attain the efficient photosensitization of fungal targets [36,37,47]. For this purpose, quaternized ammonium tetra- and octasubstituted phthalocyanines **1** (Figure 4—green) and **2** (Figure 4—blue) were synthesized [39] and tested against the conidia of *Fusarium oxysporum*, taken as the fungal pathogen model. aPDT efficiency was estimated as the logarithmic reduction in the concentration of viable conidia (Figure 2) and variation in the concentration of viable conidia (Figure 3).

Irradiation in the absence of PS did not induce a decrease in the concentration of viable *F. oxysporum* conidia; similarly, no decrease was observed in the absence of light and the presence of PS (LC, DC **1** and DC **2**, Figure 2). After 60 min of irradiation (light dose of 486 J·cm^−2^), with concentrations of 5 and 10 µM, the inactivation caused by either PS **1** or PS **2** was negligible (<1 log_10_; Figure S4). In order to attain complete inactivation, higher concentrations of PS (20, 40, and 60 μM) were tested. In this case, with the same irradiation time and 40 or 60 μM of PS **1,** it was possible to reduce the concentration of viable spores to the method’s quantification limit, taking this result as complete inactivation (Figure 2). With 20 μM and the equivalent energy dose, the inactivation corresponded to a 2 log_10_ reduction in the concentration of viable conidia. In the case of PS **2** (Figure 2), an even higher concentration may be required, since the concentrations of 20, 40, or 60 μM caused reductions of 2, 3, and 5 log_10_ in the concentration of viable conidia, respectively, with a light dose of 486 J·cm^−2^. Overall, our results confirmed that ammonium PSs above concentrations of 40 μM could efficiently inactivate *F. oxysporum* conidia.

In PS **1**, despite being considered an antimicrobial agent, the introduction of ammonium substituents to the β-position seemed to reduce the antifungal activity when compared with phthalocyanine with the same group in the α-position (complete inactivation of *C. albicans* with 1.46 μM and a light dose of 27 J·cm^−2^) [48]. When compared to other ammonium phthalocyanines with four and eight charges, the increase in charge number in PS **1** (12 charges) increased the minimal concentration needed to achieve a minimal 4 log_10_ inactivation of *C. albicans* (from 0.5, 1, 10, and 20 μM to 40 μM) with a light dose of 30 J·cm^−2^ [49]. On the other hand, the concentration of PS **2** (24 charges) needed to increase to 60 μM to achieve the same effect. However, when compared with other [50] ammonium phthalocyanines (100 μM, 10 J·cm^−2^), the synthesized PSs **1** and **2**, tested in the present study, seemed more effective. This is the first study concerning the use of ammonium phthalocyanines against *F. oxysporum* conidia. 

In order to better compare the photoinactivation efficiency of PSs **1** and **2,** the effect of the energy dose was assessed by testing two exposure periods: 30 min corresponding to an energy dose of 243 J·cm^−2^, and 60 min corresponding to 486 J.cm^−2^. The results show that the tetrasubstituted PS **1** caused the complete inactivation of *F. oxysporum* conidia (>5 log_10_ reduction, with 40 or 60 μM) with the lowest energy dose (243 J·cm^−2^); with the same energy dose, the octasubstituted PS **2** caused a 2 log_10_ reduction (60 μM). These differences in efficiency could have been due to the difference in ^1^O_2_ quantum yields (Φ_Δ_ **1** > Φ_Δ_ **2**) [39]. So, the increase in the number of charges did not seem to inherently improve photodynamic efficiency. Besides the difference in ^1^O_2_ generation, PS **2** seemed to aggregate, as seen in the absorption spectrum (Figure 1); for that reason, a decrease in the photodynamic efficiency may have resulted. 

Experiments involving the inactivation of a Gram-negative bacterial model (*Escherichia coli*) also indicated that PS **1** was more effective than PS **2** [39]. Thus, although the photosensitization of fungal structures requires higher PS concentrations, the cellular targets of photosensitization with PS **1** may be sufficiently diverse to support the prospect of a broad-spectrum (multiorganism) phytosanitary approach applicable to plant nurseries. The effectiveness of the two PSs against pathogenic fungal microbes in vitro showed their promising application in an in vivo approach, such as the one presented by Plaetzer and coworkers, who used strawberry leaves and solar light as a green irradiation source [51].

## 4. Materials and Methods

### 4.1. Synthesis and Photophysical Characterization of the Photosensitizers

The structures of the cationic PSs with ammonium groups (**1** and **2**) are depicted in Figure 4.

Phthalocyanines **1** and **2** were prepared according to previously described experimental procedures [39], using reagents with high-level purity (purchased from Merck, Steinheim, Germany). Analytical TLC was carried out on precoated silica gel sheets (Merck, 0.2 mm, Darmstadt, Germany). According to the literature, solvents were used as received or distilled and dried using standard procedures [52]. ^1^H and ^19^F NMR spectra were recorded on a Bruker Avance-300 spectrometer (Wissembourg, France) at 300.13 and 282.38 MHz, where tetramethylsilane (TMS) was used as an internal reference. Absorption and steady-state fluorescence spectra were recorded using a Shimadzu UV-2501PC (Shimadzu, Kyoto, Japan) and a Horiba Jobin-Yvon FluoroMax Plus spectrofluorometer (Horiba Ltd., Kisshoint, Japan), respectively. The absorption and emission spectra of PS **1** and **2** were measured in DMF in 1 × 1 cm quartz optical cells at 298.15 K. The fluorescence quantum yield (Φ_F_) of **1** and **2** were calculated in DMF by comparing the area below the corrected emission spectra using ZnPcF_16_ as the standard (Φ_F_ = 0.04 in acetone) [53].

### 4.2. Photosenstizer Stock Solutions

The stock solutions of PS at 500 µM were prepared in DMF or dimethyl sulfoxide (DMSO) for photophysical analyses or photodynamic inactivation assays, respectively, stored in the dark and previously sonicated for 30 min to each assay.

### 4.3. Light Source

All photodynamic inactivation assays were performed by exposing the samples and light controls to a white light (400–800 nm) from a compatible fiber-optic probe attached to a 150 W quartz/halogen lamp (model LC122, LumaCare™ MBG Technologies Inc., New Port Beach, CA, USA) with an irradiance of 135 mW·cm^–2^, measured with a Coherent FieldMaxII-Top energy meter combined with a Coherent PowerSensPS19Q energy sensor.

### 4.4. Preparation of Stock Suspensions of Fusarium oxysporum Conidia

Cultures of *Fusarium oxysporum* grown, for 7 days at 25 °C in Potato Dextrose Agar (PDA, Merck, KGaA, Darmstadt, Germany ), were used to prepare the conidia suspension as described in the literature [36]. The absence of hyphae in the suspensions was checked via light microscopy (Leitz Laborlux K, Ernst Leitz GmbH, Wetzlar, Germany). The concentration of the viable conidia was determined with the serial dilutions of an aliquot in phosphate saline buffer (PBS, pH 7.4) and spread-plated on Rose Bengal Chloramphenicol Agar (Merck, KGaA, Darmstadt, Germany). Colonies were counted after 2 days of incubation at 25 °C, and the concentration of conidia is expressed as colony forming units per milliliter (CFU·mL^−1^) of suspension.

### 4.5. Photodynamic Inactivation Assay

The photoinactivation assays were performed on PBS suspensions containing approx. 4 × 10^5^ CFU·mL^−1^ in the presence of final concentrations of 20, 40, or 60 μM of PS **1** or PS **2**. The assays were carried out in 24-well plates with a final volume of 1.5 mL. Conidia suspensions were preincubated with the PS solutions in the dark for 30 min at room temperature under stirring. After this period, irradiation was conducted for 1 h of continuous exposure. During irradiation, the suspension was kept under stirring on melting ice to prevent heating. Aliquots of 100 μL were collected at the beginning (t = 0 min), in the middle (t = 30 min), and at the end of irradiation (t = 60 min), serially diluted in PBS and spread-plated on Rose Bengal Chloramphenicol Agar in triplicate for the determination of the concentration of viable spores. Colonies were counted in the most convenient dilution after 48 h incubation at 25 °C. The average of the colonies in the replicates was used to estimate the concentration of viable conidia in the suspension and is expressed as CFU·mL^−1^. Two controls were included in each irradiation experiment: a light control (LC) submitted to the same irradiation conditions as the samples but without PS, and a dark control (DC) containing 60 μM of PS but kept in the dark. Three independent assays were conducted for each PS. The inactivation efficiency was calculated as the logarithmic (log_10_) reduction in the concentration of viable *F. oxysporum* conidia during the period corresponding to the irradiation of each independent assay.

### 4.6. Statistical Analysis

Statistical analysis was performed, and the significance of the *F. oxysporum* conidia inactivation was assessed via a two-way univariate analysis of variance (ANOVA) model with Turkey’s multiple-comparisons post hoc test. A value of *p* < 0.05 was considered significant.

## 5. Conclusions

The relations between structural features and the efficiency of photosensitization of *F. oxysporum* conidia indicated that cationic tetrasubstituted PS **1** was more efficient than octasubstituted PS **2**, most probably because the ability to generate ^1^O_2_ species was considerably higher in the former. In this study, doubling the number of charges did not improve the photoinactivation process. The obtained results support the prospects of using these cationic phthalocyanines as new phytosanitary drugs on the basis of the photodynamic control of fungal spores.

## Data Availability

Not applicable.

## Supplementary Materials

The following supporting information can be downloaded at: https://www.mdpi.com/article/10.3390/ijms24043922/s1.
