# Supplementary material for: In Vitro Photoinactivation of Fusarium oxysporum Conidia with Light-Activated Ammonium Phthalocyanines
Source: Int J Mol Sci. 2023 Feb 15;24(4):3922. doi: 10.3390/ijms24043922 (PMC9966838; doi:10.3390/ijms24043922)
Supplement: Supplementary file 1 [file ijms-24-03922-s001.zip › ijms-2182466-supplementary.pdf]

Supporting Information

In Vitro Photoinactivation of *Fusarium oxysporum* Conidia by Light-Activated Ammonium Phthalocyanines

Sara R.D. Gamelas [1], Isabel N. Sierra-Garcia [2], Augusto C. Tomé [1], Ângela Cunha [2,*] and Leandro M.O. Lourenço [1,*]

[1] LAQV-REQUIMTE, Department of Chemistry, University of Aveiro, 3810-193 Aveiro, Portugal.

[2] CESAM, Department of Biology, University of Aveiro, 3810-193 Aveiro, Portugal.

* Correspondence: acunha@ua.pt (A.C.); leandrolourenco@ua.pt (L.M.O.L.)

Contents

1. NMR spectroscopy 2
2. Photosensitization of *Fusarium oxysporum* conidia at low PS concentrations 5

1. NMR spectroscopy

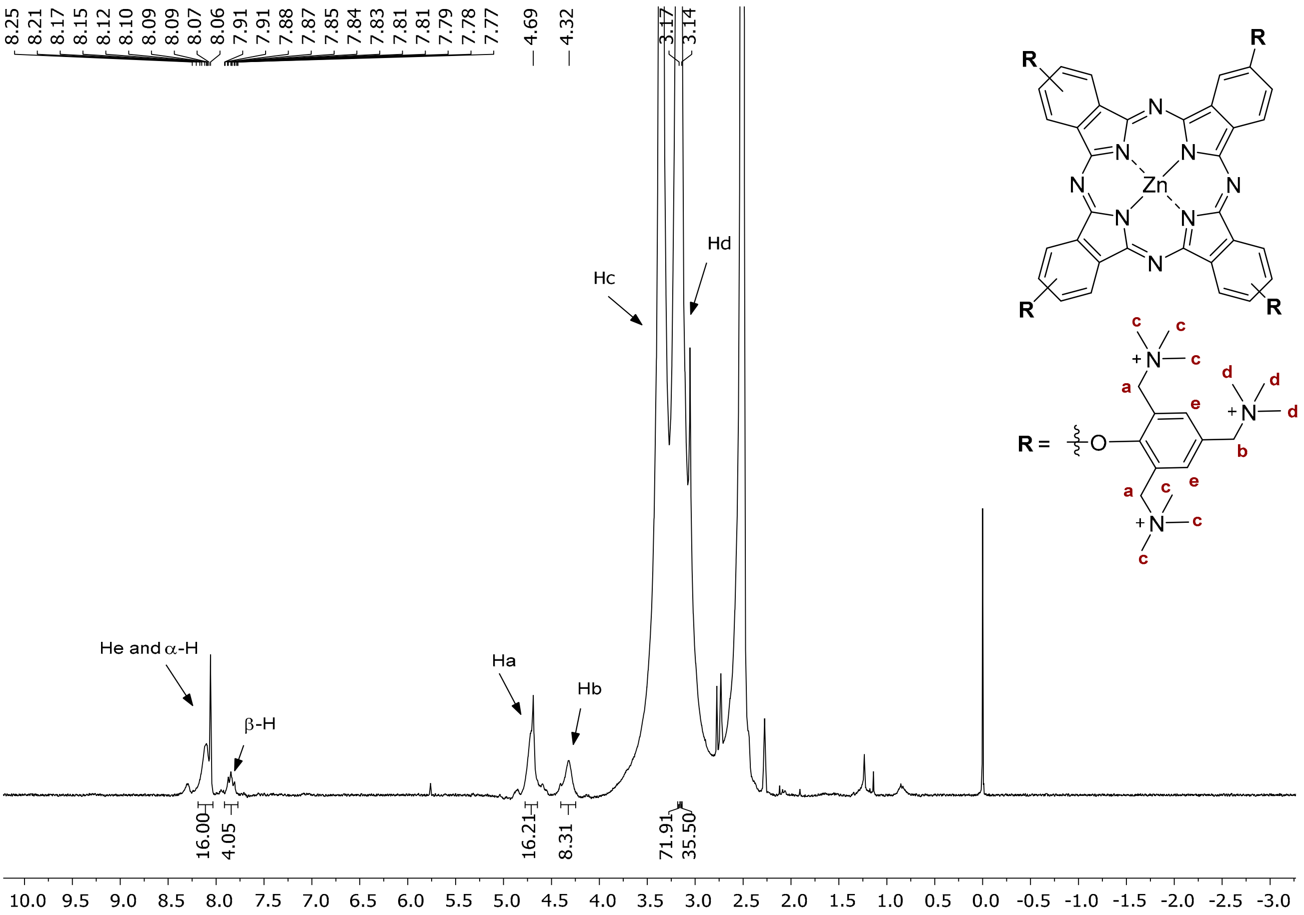

Figure S1. ^{1}H NMR (300.13 MHz) spectrum of PS **1** in DMSO-d_6.

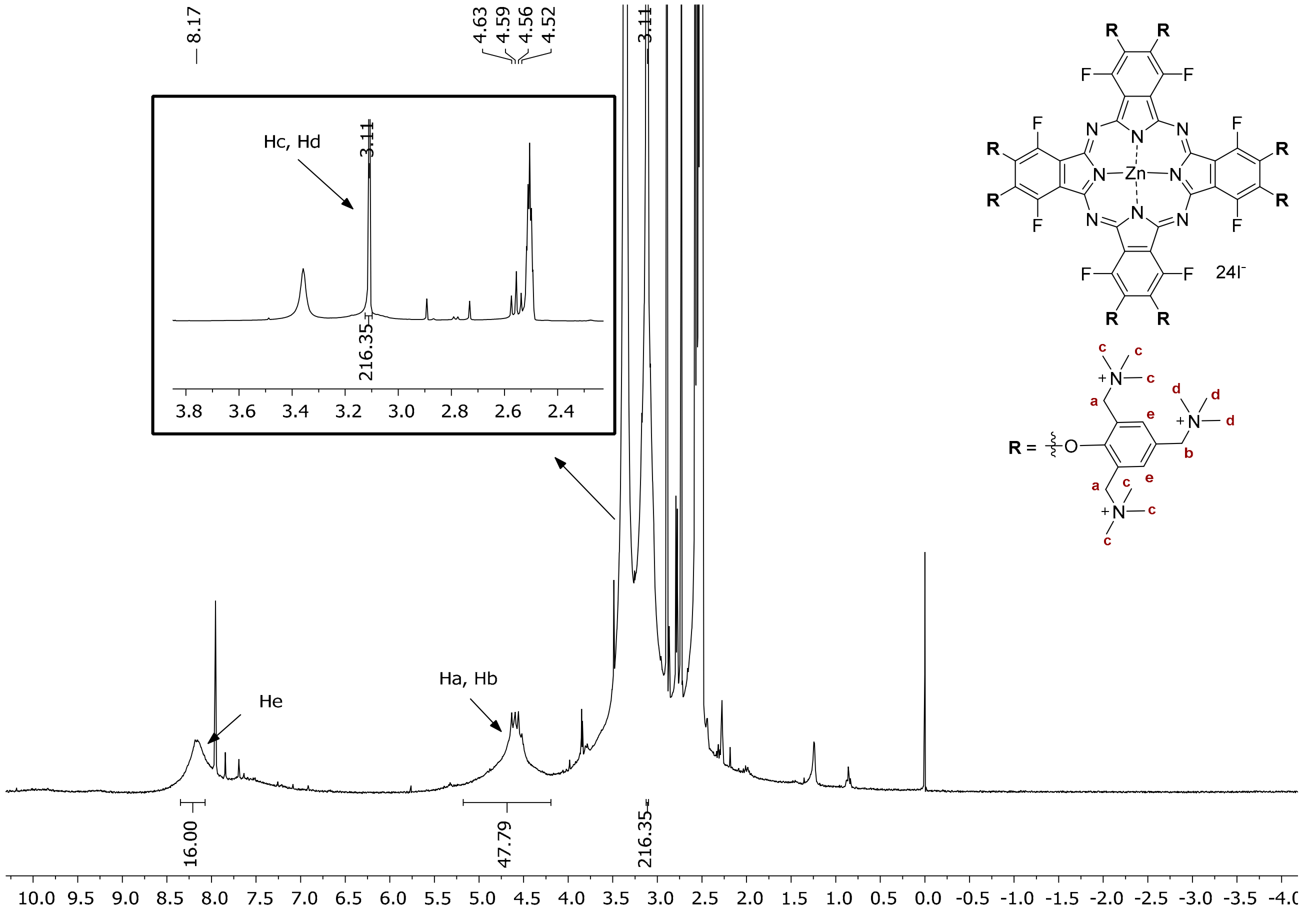

Figure S2. ^{1}H NMR (300.13 MHz) spectrum of PS **2** in DMSO-d_6.

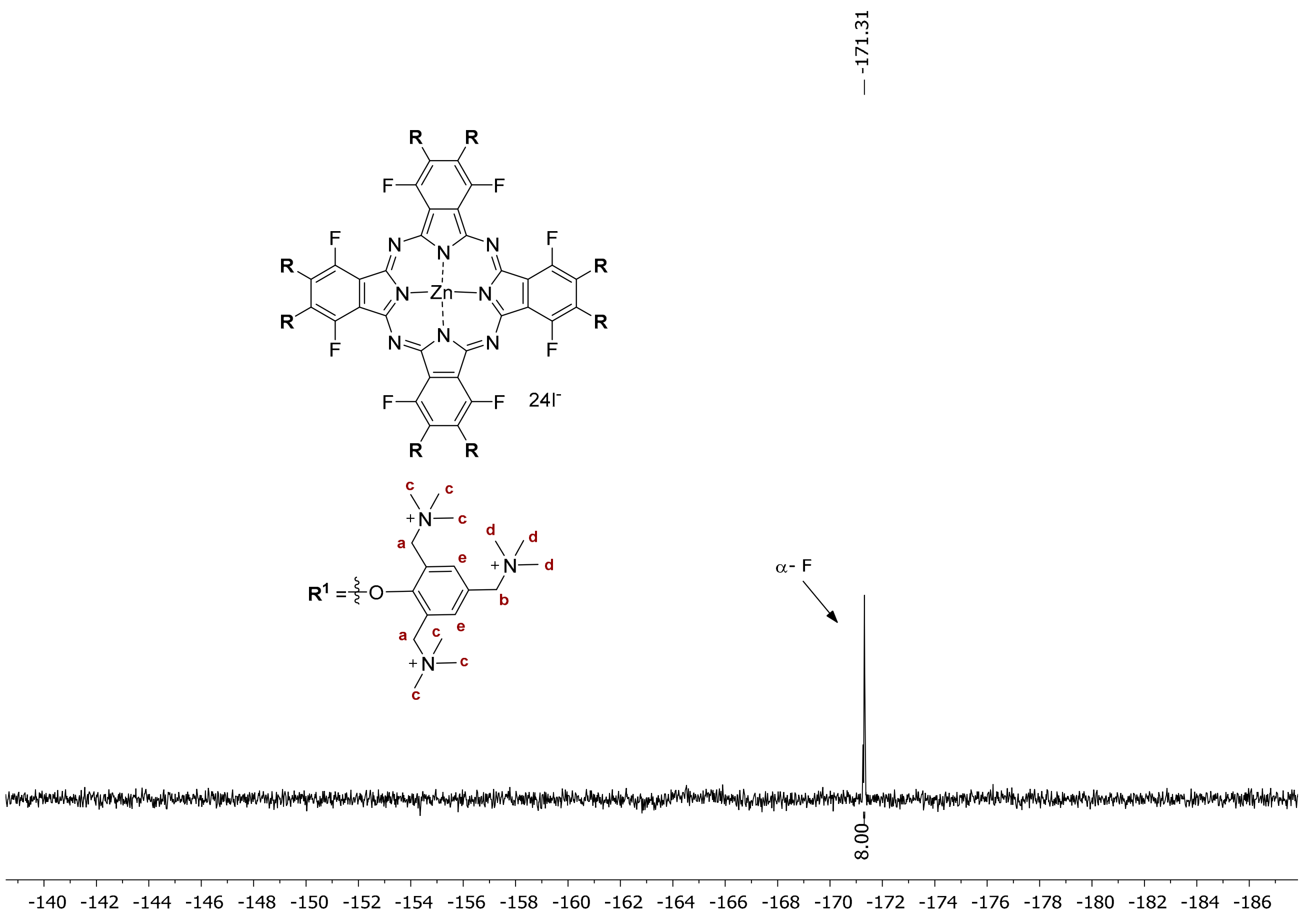

Figure S3. ^{19}F NMR (282.38 MHz) spectrum of PS **2** in DMSO-d_6.

2. Photosensitization of *Fusarium oxysporum* conidia at low PS concentrations

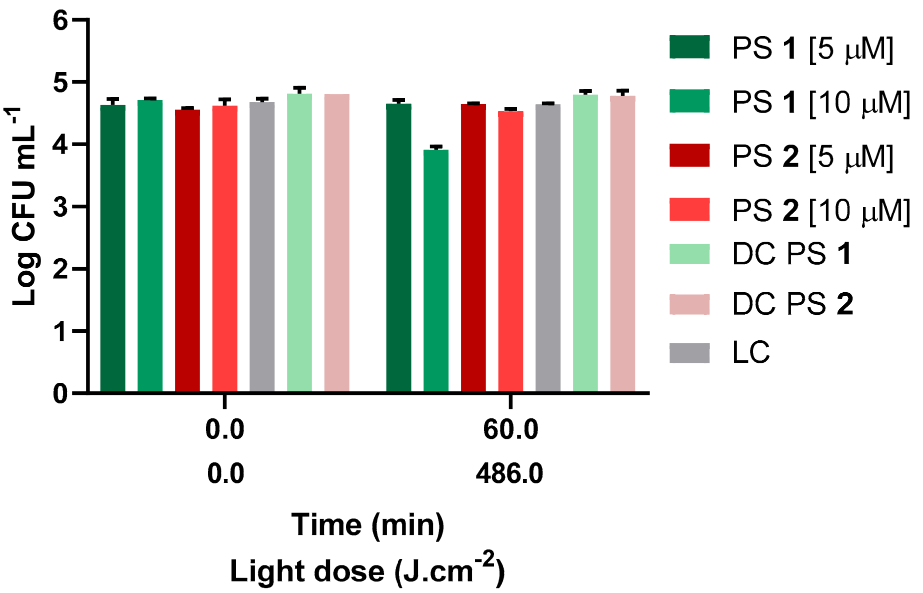

Figure S4. Variation of the concentration of viable conidia of *Fusarium oxysporum* in presence of the PS **1** and **2** at low concentrations (5 and 10 µM), exposed under white light irradiation for 60 min (irradiance of 135 $mW.cm^{-2}$, light dose of 486 $J.cm^{-2}$). LC – light control; DC PS **1** – dark control with PS **1**; DC PS **2** – dark control with PS **2**. Values are the mean of three replicate counts. Error bars represent the standard deviation.
